# Manual Physiotherapy Combined with Pelvic Floor Training in Women Suffering from Stress Urinary Incontinence and Chronic Nonspecific Low Back Pain: A Preliminary Study

**DOI:** 10.3390/healthcare10102031

**Published:** 2022-10-14

**Authors:** Gianluca Giordani, Sara De Angelis, Annunziata Isabella Parisi, Andrea Cosimo D’amico, Moira Di Re, Chiara Liumbruno, Federica Tamburella, Danilo Lisi, Giovanni Galeoto, Marco Tramontano

**Affiliations:** 1 Santa Lucia Foundation IRCCS, 00174 Rome, Italy; 2 UOC Risk Management, Azienda Ospedaliera Rilievo Nazionale Sant’Anna e San Sebastiano, 81100 Caserta, Italy; 3 Department of Neurosciences, Sapienza University of Rome, 00100 Rome, Italy

**Keywords:** stress urinary incontinence, nonspecific low back pain, pelvic floor rehabilitation, physiotherapy, women

## Abstract

Stress urinary incontinence (SUI) represents one of the most common subtypes of urinary incontinence (UI) reported by women. Studies have shown an association of SUI with nonspecific low back pain (NSLBP). The primary aim of the present study was to explore the long-term effects of a combined treatment of manual techniques and pelvic floor muscle (PFM) training in women suffering from SUI associated with NSLBP. The secondary aim was to evaluate which manual approach combined with PFM rehabilitation is more effective in improving symptoms related to SUI and in reducing pain perception related to NSLBP. Twenty-six patients suffering from SUI associated with chronic NSLBP were randomly assigned to one of two groups: the postural rehabilitation group (PRg) or the spinal mobilization group (SMg). Both groups performed a manual approach combined with PFM rehabilitation. All patients were evaluated before the treatment (T0), after 10 sessions (T1) and after 30 days from the end of the treatment (T2). The results showed an improvement in both groups in all of the investigated outcomes. Combining manual therapy and PFM training within the same therapy session may be useful for improving both SUI and NSLBP and increasing the quality of life of women suffering from SUI associated with NSLBP.

## 1. Introduction

Urinary incontinence (UI) is a common health condition in the female population. Although its prevalence increases with age, women of all ages could be affected [1,2].

Stress urinary incontinence (SUI) is defined by the International Continence Society (ICS) as “the complaint of any involuntary loss of urine on effort or physical exertion (e.g., sporting activities) or on sneezing or coughing” [3]. It represents one of the most common subtypes of UI reported by women [2], and it is typically associated with small and momentary leakages that come to an end once the intra-abdominal pressure decreases [4]. Factors such as age, pregnancy, childbirth, and hormone-related conditions have been reported to increase their prevalence [5]. It is related to poor quality of life with negative effects on different dimensions of everyday life including social activities and mental health. Moreover, in addition to having a substantial impact on the health-related quality of life, it is associated with a high level of individual and societal expenditure [6].

Several epidemiological studies have shown an association of SUI with nonspecific low back pain (NSLBP) demonstrating, moreover, that the presence of one condition may predispose the patient to the onset of the other [5,7,8,9,10,11,12].

Low back pain (LBP) is one of the most common musculoskeletal conditions in industrial countries [5,13]. It has been defined as pain, discomfort, muscle tension, or stiffness localized below the costal margin and above the inferior gluteal folds, with or without leg pain [14,15,16,17]. The most common form of LBP is non-NSLBP, a condition characterized by no known pathoanatomical causes [14,18]. Based on the duration of the symptomatology, LBP is defined as acute LBP (ALBP) when the LBP episode persists for fewer than 6 weeks, subacute LBP (SLBP) if the condition persists between 6 and 12 weeks and it is defined as chronic LBP (CLBP) when the symptoms persist for more than 3 months or longer than the expected recovery period [13,17]. Specifically, CLBP represents one of the principal causes of disability worldwide with a high impact on the quality of life [13]. Clinical practice guidelines for treating LBP provide recommendations for physical rehabilitative treatment [13,19]. Different techniques, physical exercises and rehabilitation delivery methods [20,21,22,23,24] have been developed and proved to be efficacious in improving LBP-related disorders, but to date, it has been difficult to affirm the superiority of one approach compared to another [13,19].

Although the mechanism in the development of NSLBP is not fully understood, it could be considered to be associated with changes in the trunk’s muscle control, particularly the reduced postural activity of the diaphragm, transversus abdominis and pelvic floor muscles (PFMs), which seem to be related to impairment in the spine’s mechanical support, favoring the onset of LBP [25,26]. The crucial role played by PFMs, as an integral part of both trunk and lumbo-pelvic stability as well as in the maintenance of urinary continence, leads PFM dysfunction to be associated both with SUI and NSLBP [5,27]. Thus, PFM rehabilitation may be considered an effective approach in patients with chronic NSLBP and UI [5]. On the other hand, the muscles responsible for spine stability also play an important role in the maintenance of continence [28]. Indeed, manual techniques focused on spinal mobility and postural stability could represent a valid approach both for SUI and NSLBP [29,30]. To the best of our knowledge, there are no studies investigating the short- and long-term effects of different manual technique modalities combined with PFM rehabilitation on NSLBP and UI simultaneously.

In this context, the primary aim of the present study was to explore the long-term effects of a combined treatment of manual techniques and PFM rehabilitation in women suffering from SUI associated with NSLBP. The secondary aim was to evaluate which manual approach combined with PFM rehabilitation is more effective at improving symptoms related to SUI and in reducing pain perception related to NSLBP.

## 2. Materials and Methods

### 2.1. Study Design

**Figure 1:**
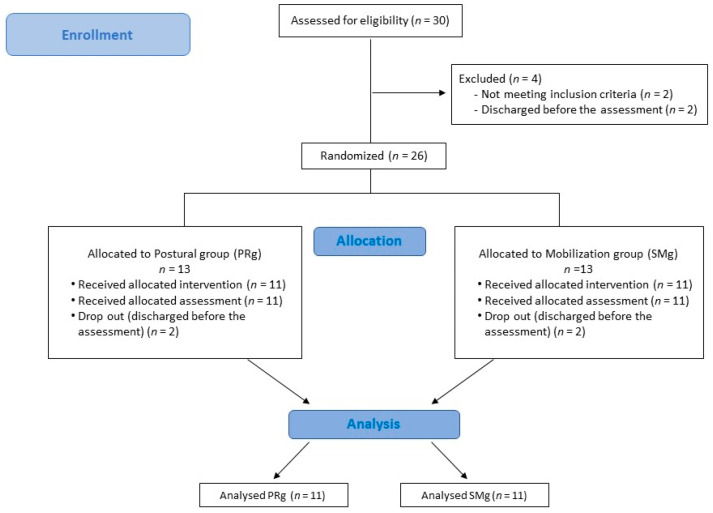
Flow diagram of the study design.

This study was a two-arm, single-blind randomized controlled trial (Figure 1).

The Consolidated Standards of Reporting Trials (CONSORT) [31] were followed. This trial was approved by the Local Ethics Committee of Fondazione Santa Lucia (FSL) with protocol number: CE/PROG.593. Participants provided their written informed consent for participation.

A researcher who was not involved in the intervention sessions assessed the patients’ eligibility to participate based on the inclusion and exclusion criteria.

### 2.2. Participants

Twenty-six patients (mean age = 54.0 ± 11.2; age range = 31–70) with a diagnosis of SUI associated with chronic NSLBP were recruited and enrolled through the databases of Fondazione Santa Lucia (FSL) based on consecutive sampling at the FSL Institute for Research and Health Care, from September 2020 to July 2021.

The inclusion criteria were women aged 18–75 years with a diagnosis of SUI (medical diagnosis) associated with nonspecific chronic LBP (medical diagnosis). The exclusion criteria were severe pelvic organs prolapse (second stage or higher following the International Continence Society (ICS) classification scheme) [32]; pregnancy; perineal denervation; inverted perineal command; presence of pelvic pain; fecal incontinence; vaginal infections; associated pathologies involving the central nervous system (CNS); psychotic disorders. All criteria were determined based on medical assessments and diagnoses. All participants did not attend physiotherapy treatment in the 6 months prior to enrollment. Furthermore, the enrolled patients did not take any medicine that could influence the clinical assessments (e.g., NSAIDs) during the period under investigation. The demographic characteristics of the sample are reported in Table 1.

### 2.3. Interventions

Two different protocols were designed: one based on the postural rehabilitation associated with perineal exercises (PRg) and the other one based on spinal mobilizations (Table 2) associated with perineal exercises (SMg). For both interventions, each session lasted 60 min. Each 60 min session consisted of 20 min of pelvic floor muscle rehabilitation and 40 min of postural rehabilitation or spinal mobilization, based on the allocation group. Both groups performed 10 sessions of the allocated intervention, organized 2 times a week for 5 weeks. All of the proposed exercises for both groups were carried out by the same experienced physiotherapist.

#### 2.3.1. Postural Rehabilitation

**Figure 2:**
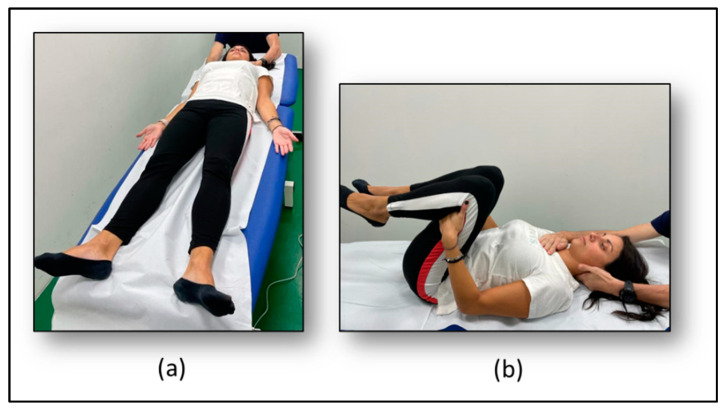
Postural rehabilitation intervention exercises. (**a**) Postural rehabilitation first posture; (**b**) Postural rehabilitation second posture.

Each session of the experimental approach was composed of 40 min of postural exercise. The postural rehabilitation intervention included specific exercises that promote proper alignment by increasing the efficiency of dynamic movement and limiting muscle imbalance and overcompensation. Participants were asked to maintain two different postures to stretch both the anterior and posterior muscle chains. The first posture (Figure 2a) consisted of lying on the back maintaining the extension of the legs to release the respiratory diaphragm and stretch the anterior muscle chain (i.e., diaphragm, pectoralis minor, scalene, sternocleidomastoid, intercostalis, iliopsoas, muscles of the arms and forearms, and hand flexors). For the second posture (Figure 2b), participants were asked to lie down on their back with their legs flexed to stretch the posterior chain (i.e., upper trapezius, levator scapulae, suboccipital, erector spinae, gluteus maximus, ischiotibial, triceps surae, and foot intrinsic muscles). For each posture, the physical therapist used verbal commands and manual contact to maintain alignment, make the necessary postural corrections to optimize the stretching and to discourage compensatory movements [36,37].

#### 2.3.2. Spinal Mobilizations

**Figure 3:**
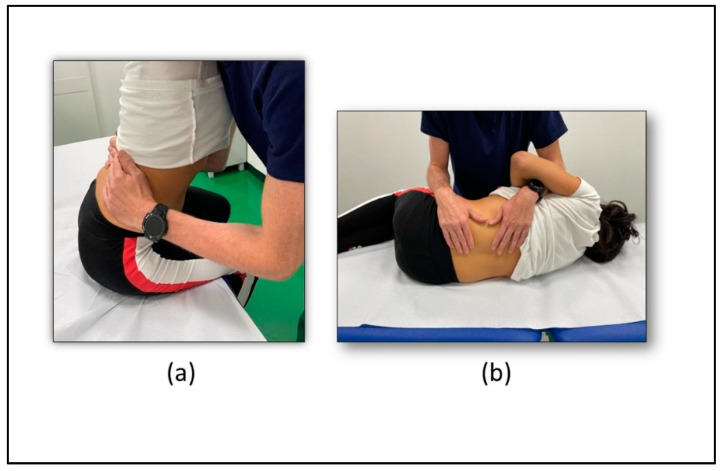
Spinal mobilization intervention exercises. (**a**) Spinal mobilization exercise 1; (**b**) Spinal mobilization exercise 2.

The conventional approach consisted of 40 min of thoracolumbar spine mobilization. Two different mobilizations were carried out. In the first one (Figure 3a), the patient was in a sitting position with both legs out of the bed and mobilization in the anteroposterior direction was provided by the physiotherapist; the second (Figure 3b) consisted of rotational mobilization with the patient in a lateral decubitus position [38].

**Table 2:** Spinal mobilization interventions description.

Spinal Mobilizations
**Exercise 1**
The patient is placed in a sitting position with the legs off of the table. The operator positions himself in front of the patient’s knee and grasps him at the level of the dorsal-lumbar spine to be mobilized passing his arms under the patient’s armpits. The patient rests the upper limbs on the operator’s shoulders so he can relax. The operator mobilizes the tract of the dorsal-lumbar spine of greatest interest by applying a force in the posterior–anterior direction and allowing it to return, following these movements with his own body. (Figure 3a)
**Exercise 2**
The patient is positioned in the lateral decubitus aligned with the front edge of the table, with the lower limbs flexed and the hand of the decubitus under the head; the limb opposite the decubitus is placed along the patient’s side. The operator positions himself in front of the patient, placing the cranial forearm at the level of the ribs and the caudal one at hip level; with his hands he causes the spinous processes of the lumbar vertebrae to be mobilized more. By moving the two forearms away in the direction of the longitudinal axis, the operator mobilizes the affected tract in an inclination opposite to the decubitus. By moving the forearms anteroposteriorly, the operator mobilizes the lumbar area of greatest interest in rotation. (Figure 3b)

#### 2.3.3. Pelvic Floor Muscle Rehabilitation

Perineal exercises were performed in both of the allocated approaches. The protocol consisted of two exercises: 10 min of perineal contraction and relaxation and 10 min of stretch–reflex, for a total of 20 min of perineal exercises. In the first exercise, a slow contraction for 5 s and a slow relaxation for 5 s of the perineal muscles were required. The second exercise required a slow contraction for 5 s, holding of the contraction for 5 s and a slow relaxation for 5 s. For both exercises, the rest time was double the working time, so 20 s for the first exercise and 30 s for the second. All the participants performed the same exercises.

### 2.4. Outcome Measures

At enrolment, clinical and demographic data were collected. All patients were evaluated before the treatment (T0), after 10 sessions of treatment (T1) and after 30 days from the end of the treatment (T2) in order to evaluate the prolonged effects of the treatment.

The primary outcome measure was the International Consultation on Incontinence Questionnaire-Urinary Short Form (ICIQ-UI SF) [33] to evaluate the severity of urinary loss and the quality of life (QoL). Secondary outcome measures were the pain visual analogue scale (VAS) [34] to assess LBP. Moreover, a digital assessment (bi-digital palpation) of pelvic floor muscle strength (PFS) was performed. PFM strength was graded using the modified Oxford scale, assigning a score from 0–5 [5,35]. All of the assessments were performed by a trained and experienced physiotherapist, different from the one who carried out the rehabilitative interventions.

### 2.5. Sample Size

This sample size complied with the minimum number of participants recommended by a power analysis performed on the preliminary data (α = 0.05; β = 0.8; ES = 0.5) for nonparametric between-group comparisons [39]. This sample size estimation procedure recommends that at least 10 patients be included in each group [40].

### 2.6. Blinding

A researcher not involved in the intervention sessions carried out the randomization. Block randomization was performed with a computer-generated randomization list using a block size. To ensure the concealment of the allocation for the two groups, a computer program that generates random numbers to select random permuted blocks with a block size of eight and an equal allocation ratio was used. The researcher responsible for the randomization process deposited the list in secure web-based storage.

### 2.7. Statistical Analysis

A nonparametric approach was used. The Wilcoxon signed rank test was used for the within-subject comparisons for both groups at times T0vs. 1 and T0vs. T2. The Mann–Whitney U-test was used to compare data between groups at T0, T1 and T2. The IBM SPSS Statistics software (v23, IBM Corp., Armonk, NY, USA) was used.

## 3. Results

Twenty-six patients with a diagnosis of SUI associated with chronic NSLBP met the inclusion criteria and were enrolled in the study. Four of the enrolled subjects left the study before the end for reasons not related to the study. The statistical analysis was performed using the data of twenty-two participants (PRg = 11; SMg = 11) (see Figure 1).

There were no significant differences between groups in demographics and clinical data at the baseline (T0) (Table 1).

The within-subject analysis showed a significant improvement in both groups in all the investigated outcomes (i.e., ICIQ-SF, VAS and PFS). Significant statistical differences (*p* < 0.05) were found both between the baseline and the post-treatment assessment (T1) and between the baseline and the 30 day follow-up (T2). The results of the within-subject analysis are shown in Table 3.

The between-subject analysis showed no significant difference between the two groups, neither at T1 nor at T2 (Table 4).

## 4. Discussion

The first aim of the present study was to explore the effectiveness of a combined treatment of manual techniques and PFM rehabilitation in women with SUI associated with NSLBP. Specifically, the patient’s quality of life and perception of back pain and the strength of the complex of the levator anus musculature (in particular, the pubococcygeus muscle) were assessed. The results show that both of the combined approaches to PFM rehabilitation were useful in improving SUI symptoms. Indeed, 10 sessions, two times a week for 5 weeks, led to an improvement in the quality of life in relation to incontinence and in pain perception in relation to NSLBP. The improvements were clinically significant at the end of the training and also at 1 month after the end of the treatment (i.e., follow-up).

In this study, two manual approaches (manual therapy for NSLBP and PFM rehabilitation for SUI) were combined simultaneously in the same therapeutic session to improve both conditions. Indeed, the muscles responsible for spine stability play an important role not only in trunk and lumbo-pelvic stability but also in the maintenance of continence [28]. Indeed, diaphragm superiorly, PFM inferiorly, transversus abdominis anteriorly, and deep lumbar extensor muscles posteriorly work in synergy in a complex anatomic structure with neurologically directed muscular and fascial components and a specific biomechanical function. For these reasons, the novelty of our preliminary study was to design a global manual approach that is not only focused on PFM rehabilitation but also on the spine. According to other studies, we also found a positive clinical effect in the follow-up, 1 month after the end of the training. Indeed, a previous study [5] reported that PFM rehabilitation alone produces only short-term effects on SUI, and 6–12 weeks of training more than three times a week sessions are recommended for long-term effects.

Furthermore, as previously reported [41], a program that combines pelvic floor muscle exercises with low-load core stability awareness exercises (i.e., abdominal draw-in, heel slide, and heel off exercises) may be helpful in reducing the amount and frequency of UI as well as in improving the QoL for women with LBP who have SUI.

We can hypothesize that a global approach leading to an improvement in the musculoskeletal system and spine stability can produce long-lasting effects on NSLBP and SUI. The secondary aim of this study was to evaluate which manual approach combined with PFM rehabilitation was more effective at improving SUI and reducing pain perception related to NSLBP. Our results showed no clinically significant differences between groups at the end of the training and at follow-up. Although the two proposed manual approaches were different from each other, one was more global and active and one more selective and passive. Reasonably, both treatments play a role in improving trunk muscle stability. Indeed, according to previous studies [26,27], trunk stability has a role in reducing pain and disability in chronic LBP and related symptoms in SUI.

### Limitations

We acknowledge some of the limitations of this study. First, the sample size was relatively small, and this could have affected the statistical analysis. Another important limitation was the use of digital palpation in the PFM strength assessment. Although currently it has been used in clinical practice and no evaluation tool is considered the golden standard [42], many researchers consider digital palpation unreliable, subjective and not sensitive [35]. On the other hand, different studies have shown a correlation between digital palpation and other methods and consider it an objective assessment tool [43]. Thus, because this reproducibility remains questionable, instruments such as biofeedback monitors, manometers, perineometers and dynamometers may be considered for use in future studies as support in the PFM strength assessments.

## 5. Conclusions

In conclusion, combining manual therapy and PFM rehabilitation within the same therapy session may be useful for improving both SUI and NSLBP symptoms and for increasing the quality of life in women suffering from SUI associated with NSLBP.

## Figures and Tables

**Table 1:** Demographic and clinical characteristics at the baseline of the trial.

	PRg (*n* = 11)	SMg (*n* = 11)	*p*-Value
Age (years) ± SD	49.6 ± 11.3	58.5 ± 9.6	0.063
Age (years)			
31–40	1	3	
41–50	0	0	
51–60	5	5	
61–70	5	3	
Time since NSLBP diagnosis (months) ± SD	1.5 ± 2.3	2.0 ± 2.1	0.567
Time since SUI symptoms onset (years) ± SD	4.9 ± 2.9	4.4 ± 2.8	0.673
ICIQ-SF	9.6 ± 1.6	8.1 ± 3.1	0.332
VAS	4.9 ± 1.6	5.7 ± 1.5	0.171
Pelvic floor strength	1.7 ± 0.8	2.2 ± 0.8	0.332

SD = standard deviation. PRg = postural rehabilitation group; SMg = spinal mobilizations group; NSLBP = nonspecific low back pain; SUI = stress urinary incontinence; ICIQ-SF = International Consultation on Incontinence Questionnaire-Urinary Short Form, scored from 0 (no leakage of urine and no effect on quality of life) to 21 (greatest severity of symptoms and effect on quality of life) [33]; VAS = visual analogue scale, ranging from 0 (least pain) to 10 (greatest pain) [34]; PFS = pelvic floor strength, graded by the modified Oxford scale which ranges from 0 (no discernible pelvic floor muscle contraction) to 5 (strong pelvic floor muscle contraction) [35]. The *p*-value was significant at *p* < 0.05.

**Table 3:** Within-subject analysis results.

	PRg					SMg				
	T0	T1	T2	T0 vs. T1	T0 vs. T2	T0	T1	T2	T0 vs. T1	T0 vs. T2
ICIQ-SF ^1^	9.6 ± 1.6	7.1 ± 3.9	6.5 ± 4.2	0.032 *	0.026 *	8.1 ± 3.1	6.1 ± 3.9	5.4 ± 3.5	0.042 *	0.036 *
VAS ^1^	4.9 ± 1.6	2.5 ± 1.9	2.1 ± 2.3	0.005 *	0.013 *	5.7 ± 1.5	1.3 ± 1.5	2.1 ± 2.8	0.003 *	0.006 *
PFS^1^	1.7 ± 0.8	3.1 ± 0.9	3.4 ± 1.0	0.006 *	0.003 *	2.2 ± 0.8	3.6 ± 1.4	3.4 ± 1.4	0.011*	0.041 *

^1^ Mean ± standard deviation of a clinical scale’s scores at T0, T1 and T2. PRg = postural rehabilitation group; SMg = spinal mobilization group; ICIQ-SF = International Consultation on Incontinence Questionnaire-Urinary Short Form, scoring from 0 (no leakage of urine and no effect on quality of life) to 21 (greatest severity of symptoms and effect on quality of life) [33]; VAS = visual analogue scale, ranging from 0 (least pain) to 10 (greatest pain) [34]; PFS = pelvic floor strength, graded by the modified Oxford scale which ranges from 0 (no discernible pelvic floor muscles contraction) to 5 (strong pelvic floor muscles contraction) [35]. * Significant at *p* < 0.05.

**Table 4:** Between-subject analysis results.

	PRg			SMg			PRg vs. SMg	
	T0	T1	T2	T0	T1	T2	Comparison at T1	Comparison at T2
ICIQ-SF ^1^	9.6 ± 1.6	7.1 ± 3.9	6.5 ±4.2	8.1 ± 3.1	6.1 ± 3.9	5.4 ± 3.5	0.478	0.519
VAS ^1^	4.9 ± 1.6	2.5 ± 1.9	2.1 ± 2.3	5.7 ± 1.5	1.3 ± 1.5	2.1 ± 2.8	0.133	0.797
PFS ^1^	1.7 ± 0.8	3.1 ± 0.9	3.4 ±1.0	2.2 ± 0.8	3.6 ± 1.4	3.4 ± 1.4	0.270	0.949

^1^ Mean ± standard deviation of a clinical scale’s scores at T0, T1 and T2. PRg = postural rehabilitation group; SMg = spinal mobilization group; ICIQ-SF = International Consultation on Incontinence Questionnaire-Urinary Short Form scoring from 0 (no leakage of urine and no effect on quality of life) to 21 (greatest severity of symptoms and effect on quality of life) [33]; VAS = visual analogue scale, ranging from 0 (least pain) to 10 (greatest pain) [34]; PFS = pelvic floor strength, graded by the modified Oxford scale which ranges from 0 (no discernible pelvic floor muscles contraction) to 5 (strong pelvic floor muscles contraction) [35].

## Data Availability

The datasets used and/or analyzed during the current study are available from the corresponding author upon reasonable request.
